# *HER2* Status in Colorectal Cancer: Its Clinical Significance and the Relationship between *HER2* Gene Amplification and Expression

**DOI:** 10.1371/journal.pone.0098528

**Published:** 2014-05-30

**Authors:** An Na Seo, Yoonjin Kwak, Duck-Woo Kim, Sung-Bum Kang, Gheeyoung Choe, Woo Ho Kim, Hye Seung Lee

**Affiliations:** 1 Department of Pathology, Kyungpook National University Hospital, Kyungpook National University School of Medicine, Daegu, Republic of Korea; 2 Department of Pathology, Seoul National University College of Medicine, Seoul, Republic of Korea; 3 Department of Surgery, Seoul National University Bundang Hospital, Seongnam-si, Republic of Korea; 4 Department of Pathology, Seoul National University Bundang Hospital, Seongnam-si, Republic of Korea; Baylor College of Medicine, United States of America

## Abstract

This study aimed at determining the incidence and clinical implications of *HER2* status in primary colorectal cancer (CRC). *HER2* status was investigated in two retrospective cohorts of 365 consecutive CRC patients (cohort 1) and 174 advanced CRC patients with synchronous or metachronous distant metastasis (cohort 2). *HER2* status was determined by performing dual-color silver in-situ hybridization (SISH), mRNA in-situ hybridization (ISH), and immunohistochemistry (IHC). The incidence of HER2 protein overexpression (IHC 2+/3+) was approximately 6% (22 of 365 in cohort 1; 10 of 174 in cohort 2). *HER2* gene amplification was observed in 5.8% of the patients from cohort 1 and 6.3% of the patients from cohort 2. *HER2* gene amplification was more frequently observed in CRCs located in the rectum than in the right and left colon (*P* = 0.013 in cohort 1; *P* = 0.009 in cohort 2). *HER2* status, determined by IHC, ISH, and dual-color SISH, was not significantly associated with aggressive CRC behaviour or patients' prognosis in both the cohorts. Of the combined cohort with a total of 539 cases, the concordance rate was 95.5% between dual-color SISH and IHC detection methods. On excluding equivocally immunostained cases (IHC 2+), the concordance rate was 97.7%. *HER2* mRNA overtranscription, detected by ISH, significantly correlated with protein overexpression and gene amplification (*P*<0.001). *HER2* gene amplification was identified in a minority of CRC patients with high concordance rates between dual-color SISH and IHC detection methods. Although *HER2* status did not predict patients' prognosis, our findings may serve as a basis for future studies on patient selection for *HER2* targeted therapy.

## Introduction

Despite advances in surgical techniques and adjuvant chemotherapeutic regimens, colorectal cancer (CRC) remains one of the major leading causes of cancer-related deaths world over [1], [2]. Further improvements in understanding tumor biology and identifying oncogenic drivers have led to the development of new therapeutic targets [2], [3]. Therefore, identification of biological markers for targeted therapy continues to be a high priority in human cancer treatment [4]. Anti-epidermal growth factor receptor (EGFR) monoclonal antibody treatment, including that with cetuximab and panitumumab, has been reported to improve progression-free survival in advanced CRC patients with wild-type *KRAS* [5]–[7]. Despite the undeniable therapeutic progress, a considerable proportion of cancer patients respond poorly to therapy, and therapeutic response cannot be exactly predicted. Therefore, it is of great interest to identify molecular biomarkers for predicting outcome, therapeutic response, and potential therapeutic targets in CRC patients.

The human epidermal growth factor receptor 2 (HER2) is a transmembrane receptor tyrosine kinase, which is a member of the EGFR family [8], [9]. Activation of *HER2* plays a key role in cell proliferation, cell differentiation, inhibition of apoptosis, and tumor progression [8]–[10]. Trastuzumab, a humanized monoclonal antibody, targets the extracellular domain of the *HER2* receptor. Its therapeutic benefit has been demonstrated in *HER2*-positive breast cancer patients [11], [12]. In addition, one landmark study in 2010 reported that the combination of trastuzumab with conventional chemotherapy significantly improved patients' survival in patients with *HER2*-positive advanced gastric or gastro-esophageal junction cancer [13]. *HER2* gene amplification and/or protein overexpression were observed in 15–20% of breast cancer cases and were correlated with aggressive phenotype, metastases, and adverse clinical outcome [14], [15]. On the other hand, *HER2* gene amplification and/or protein overexpression were detected in −22% of gastric cancer patients, but prognostic significance was controversial [16]. Because trastuzumab has significantly improved overall survival in breast and gastric cancers, it is of great clinical interest whether HER2 blockade may be a useful clinical strategy in other human cancers [17]. Although a few studies have reported the incidence and clinical implication of *HER2* status in CRC patients [18]–[22], its clinical significance has not yet been fully elucidated.

We, therefore, conducted this study to determine the incidence and clinical significance of *HER2*-positive status in consecutive primary CRC patients. Because targeted chemotherapy was applied to advanced CRC patients in daily practice and the advanced cases were not sufficiently included in consecutive cohort 1, we additionally enrolled advanced CRC patients with synchronous or metachronous distant metastasis (cohort 2). We evaluated *HER2* gene amplification, messenger RNA (mRNA) transcription, and protein expression status in the primary tumors of two retrospective cohorts, and also analyzed the concordance rates of the detection methods.

## Materials and Methods

### Patients and samples

A total of 539 CRC cases treated by radical surgery without any preoperative therapy were enrolled into the study from the Department of Pathology, Seoul National University Bundang Hospital. Cohort 1 consisted of 365 consecutive CRC patients treated between January 2005 and December 2006. Cohort 2 comprised of 174 advanced CRC patients with synchronous or metachronous distant metastasis, who had undergone surgical resections for primary CRC between May 2003 and December 2009 except cohort 1. All the patients received treatment according to standard practice guidelines following surgery, unless medically contraindicated. All the cases were reviewed by 2 pathologists (A.N.S and H.S.L) and were staged according to the 7^th^ edition of the American Joint Committee's *Cancer Staging Manual* [23]. Clinicopathological characteristics were obtained from patients' medical records and pathology reports. Follow-up information including the patient outcome and the time interval between the date of surgical resection and death was collected. The cases lost to follow-up and deaths from causes other than CRC were considered censored data for the survival analysis.

### Ethical statement

All human specimens were obtained from the files of surgically resected cases examined at the Department of Pathology, Seoul National University Bundang Hospital for the pathologic diagnosis. The retrospective study was performed using the stored samples after the pathologic diagnosis, and all of the samples were anonymized before the study. The study was approved by the Institutional Review Board of Seoul National University Bundang Hospital (reference: B-1210/174-301). The participants did not provide written informed consent in this study. The Institutional Review Board waived the need for written informed consent under the condition of anonymization and no additional intervention to the participants.

### Tissue array method

For both cohorts, surgically resected primary CRC specimens were formalin-fixed and paraffin-embedded (FFPE). These primary tumor blocks were used for the construction of tissue array blocks. Briefly, from the representative areas of the harvested blocks in each case, a single core with a diameter of 2 mm were obtained and precisely arranged into new recipient blocks using a trephine apparatus, as described previously by the author's group (Superbiochips Laboratories, Seoul, South Korea) [24]. An adequate case was defined as a tumor occupying ≥20% of the core area.

### Immunohistochemistry (IHC)

HER2 IHC was performed using PATHWAY anti-HER2/neu (4B5; rabbit monoclonal; pre-dilution; Ventana Medical Systems, Tucson, AZ, USA) antibody and ultraView Universal DAB kit (Ventana Medical Systems) on an automatic immunostainer (BenchMark XT, Ventana Medical Systems), according to the manufacturer's instructions. IHC scoring was independently performed by two pathologists (A.N.S and H.S.L) without prior knowledge of clinicopathological information or molecular results obtained via other methods. The scoring was performed according to the DAKO HercepTest™ guidelines (DAKO) for gastric cancer as follows [25]: 0, no reactivity or membrane staining in <10% of tumor cells; 1+, faint/barely perceptible partial membrane staining in ≥10% of tumor cells; 2+, weak-to-moderate complete or basolateral membrane staining in ≥10% of tumor cells; 3+, moderate-to-strong complete membrane staining in ≥10% of tumor cells. The two pathologists completely agreed on all the IHC 3+ cases, whereas final consensus was determined by discussion via the multi-head microscope in a few discrepant cases with IHC 1+ or 2+. IHC 2+ and 3+ were considered to indicate protein overexpression.

### *HER2* mRNA in situ hybridization (ISH)

For detecting *HER2* mRNA overtranscription, RNAscope 2-plex (Advanced Cell Diagnostics, Hayward, CA, USA) was performed according to the manufacturer's standard recommendations. The interpretation was performed according to the instructions in the RNA scope FFPE Assay Kit as described previously [26]: no staining (score of 0); staining in <10% of tumor cells that was difficult to identify at x40 objective lens (score of 1); staining in ≥10% of tumor cells that was difficult to identify at x20 objective lens but easy at x40 objective lens (score of 2); staining in ≥10% of tumor cells that was difficult to identify at x10 objective lens but easy at x20 objective lens (score of 3); staining in ≥10% of tumor cells that was easy to identify at x10 objective lens (score of 4). A score of 4 indicates *HER2* overtranscription. ISH was independently interpreted by two pathologists (A.N.S and H.S.L) without prior knowledge of clinicopathological information or HER2 status obtained via other methods.

### Dual-color silver *in-situ* hybridisation (SISH)

Bright-field dual-color SISH analysis was performed using the automatic SISH staining device (BenchMark XT, Ventana Medical Systems), according to the manufacturer's protocols for INFORM *HER2* DNA and INFORM Chromosome 17 (CEP17) probes (Ventana Medical Systems). *HER2*/CEP17 SISH signals were counted according to the interpretive guideline for Ventana INFORM *HER2* DNA probe staining of gastric cancer cells (Ventana Medical Systems). Tumor cells were scanned for hot spots by using 20× or 40× objectives, and the area with the highest signals was selected. The signals were counted in 20 non-overlapping tumor cell nuclei from each case using 60× or 100× objectives by two pathologists (A.N.S and H.S.L) who were blinded to HER2 status by other detection methods and clinical information. Small or large clusters were considered to be 6 signals and 12 signals, respectively. *HER2* gene amplification was defined as a *HER2*/CEP17 ratio of ≥2.0 in 20 tumor nuclei. The equivocal cases (ratio: 1.8 to 2.2) were recounted in at least 20 non-overlapping nuclei of different tumor cells at a second target area, and a new *HER2*/CEP17 ratio was recalculated. Normal colon epithelial cells and other adjacent benign cells served as the internal controls.

### Microsatellite instability (MSI)

MSI results were generated by comparison of the allelic profiles of the 5 microsatellite markers (BAT-26, BAT-25, D5S346, D17S250, and S2S123) in tumor and corresponding normal samples. The polymerase chain reaction products for FFPE tissues were analyzed using a DNA auto-sequence (ABI 3731 genetic analyzer; Applied Biosystems, Foster City, CA) according to the protocol described previously [27].

### Statistical analyses

The association between clinicopathological features and *HER2* status was analysed by using Chi-square or Fisher's exact test, and Wilcoxon/Mann-Whitney test, if appropriate. The Spearman rank correlation or Kendall's tau-b test was used to assess the correlation between the detection methods. The association between *HER2* status and overall survival was determined using the Kaplan-Meier method, and the significance of the differences between groups was compared using the log-rank test or Breslow test, if survival curves were crossed during follow-up periods. Multivariate survival analyses were performed using the Cox proportional hazards model, and the hazard ratio and its 95% confidence interval were calculated for each factor. All the tests were two-sided, with *P* value of <0.05 considered to indicate statistical significance. All the statistical analyses were performed using the IBM SPSS statistics 20 (Armonk, NY, USA) software.

## Results

### Patients' clinicopathologic characteristics

Table 1 provides demographics and baseline clinicopathological characteristics of the patients included in each cohort. Cohort 1 included 202 (55.3%) male and 163 (44.7%) female with median age of 65 years (range: 20–95 years). Additionally, among 353 patients with MSI analysis available, 321 (90.9%) had microsatellite stable (MSS) or microsatellite instability-low (MSI-L) whereas 32 (9.1%) had microsatellite instability-high (MSI-H). On the other hand, cohort 2 included 94 (54.0%) male and 80 (46.0%) female with median age of 60 years (range: 28–93 years). MSS/MSI-L was found in 159 (98.1%) and MSI-H in 3 (1.9%) out of 162 patients with MSI analysis available.

**Table 1:** Demographics and clinical characteristics of patients in each cohort.

Characteristic	Cohort 1	Cohort 2
	*N* (%)	*N* (%)
Age (years)		
Median	65.0	60.0
Range	20 to 95	28 to 93
Gender		
Male	202 (55.3)	94 (54.0)
Female	163 (44.7)	80 (46.0)
Histologic differentiation		
Low grade	331 (93.5)	149 (85.6)
High grade	23 (6.5)	25 (14.4)
Primary location		
Cecum	12 (3.3)	8 (4.6)
Ascending	54 (14.8)	17 (9.8)
Hepatic flexure	20 (5.5)	13 (7.5)
Transverse	16 (4.4)	9 (5.2)
Splenic flexure	6 (1.6)	5 (2.9)
Descending	18 (4.9)	8 (4.6)
Sigmoid	114 (31.2)	47 (27.0)
Rectum	125 (34.2)	67 (38.5)
Tumor border		
Expanding	59 (16.2)	15 (8.6)
Infiltrative	306 (83.8)	159 (91.4)
Tumor size (cm)		
Median	5.0	5.4
Range	1.0 to 13.0	2.0 to 27.0
Tumor depth (pT)		
1	14 (3.8)	1 (0.6)
2	46 (12.6)	4 (2.3)
3	238 (65.2)	102 (58.6)
4	67 (18.4)	67 (38.5)
LN metastasis		
Absent	170 (46.6)	32 (18.4)
Present	195 (53.4)	142 (81.6)
Distant metastasis at initial diagnosis		
Absent	299 (81.9)	61 (35.1)
Present	66 (18.1)	113 (64.9)
TNM stage		
I	46 (12.6)	4 (2.3)
II	118 (32.3)	17 (9.8)
III	135 (37.0)	40 (23.0)
IV	66 (18.1)	113 (64.9)
Microsatellite instability (MSI)*		
MSS/MSI-L	321 (90.9)	159 (98.1)
MSI-H	32 (9.1)	3 (1.9)
Total	365 (100.0)	174 (100.0)

Abbreviations: *N*, number; LN, lymph node; TNM, tumor-node- metastasis; MSS, microsatellite stable; MSI-L, microsatellite instability-low; MSI-H, microsatellite instability-high.

*Missing value was included.

### *HER2* status in CRC patients

**Figure 1:**
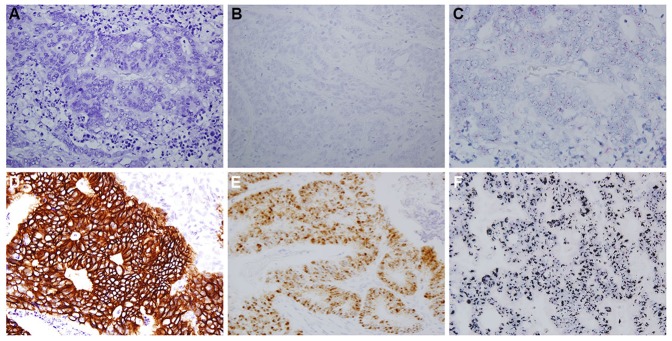
Representative figures of *HER2* status by IHC, ISH, and dual-color SISH in CRC patients. (A) IHC 0 (40× objective); (B) score 0 by mRNA ISH (40× objective); (C) *HER2* gene disomy (60× objective); (D) IHC 3+; (E) score 4 by mRNA ISH; (F) *HER2* gene amplification (*HER2*/CEP17 ratio ≥2).

The HER2 protein was faintly or weakly expressed in the membrane and/or cytoplasm of normal colonic epithelium, whereas the membrane of the tumor cells presented various staining patterns (Figure 1). In consecutive cohort 1, IHC 0 was found in 296 of 365 patients (81.1%), IHC 1+ in 47 (12.9%), IHC 2+ in 14 (3.8%), and IHC 3+ in 8 (2.2%). In cohort 2 of advanced cases, IHC 0 was observed in 139 of 174 patients (79.9%), IHC 1+ in 25 (14.4%), IHC 2+ in 5 (2.9%), and IHC 3+ in 5 (2.9%). HER2 overexpression was detected in 6.0% of cohort 1 and 5.8% of cohort 2.

A few scattered brown punctate dots were observed in the nucleus and/or cytoplasm of normal colonic epithelium by using *HER2* mRNA ISH, whereas various nuclear and/or cytoplasmic staining was observed in the tumor cells (Figure 1). In cohort 1, ISH score 0 was observed in 12 of 365 patients (3.3%), score 1 in 118 (32.3%), score 2 in 160 (43.8%), score 3 in 66 (18.1%), and score 4 in 9 (2.5%). In cohort 2, ISH score 0 was observed in 11 of 174 patients (6.3%), score 1 in 41 (23.6%), score 2 in 93 (53.4%), score 3 in 25 (14.4%), and score 4 in 4 (2.3%). *HER2* mRNA overtranscription was observed in 2.5% of cohort 1 and 2.3% of cohort 2.

*HER2* signals were detected as disomy in normal colonic epithelium, thus it was used as the internal controls. However, *HER2* signals varied throughout the tumor cells (Figure 1). The median *HER2*/CEP17 ratio was 1.16 (range: 0.68 to 19.62) in cohort 1 and 1.19 (range: 0.57 to 21.89) in cohort 2. *HER2* gene amplification was detected in 21 of 365 patients (5.8%) in cohort 1, and 11 of 174 (6.3%) in cohort 2. There was no significant difference in the incidence of *HER2* positivity between consecutive cohort 1 cases and advanced cohort 2 cases, regardless of the detection method (IHC, ISH, or SISH). Of a total of 32 amplified cases, mean *HER2* gene copy numbers (GCN) was 6 or more in 16 cases. According to the gastric cancer criteria set by the ToGA trial [13], *HER2* positivity is defined as IHC 3+ or IHC 2+ with *HER2* gene amplification. *HER2* positivity was found in 3.6% (13 of 365 patients) of cohort 1 and 4.0% (7 of 174 patients) of cohort 2. We also investigated chromosome 17 (CEP17) alterations and observed a homogeneous CEP17 signal pattern. The median CEP17 was 2.15 (range: 1.25 to 3.50) in cohort 1 and 1.95 (range: 0.83 to 3.15) in cohort 2. CEP17 polysomy, which was defined as a value of ≥3 CEP17 signals/nucleus, was observed in 2.7% (10 of 365 patients) of cohort 1 and 2.3% (4 of 174 patients) of cohort 2.

### The concordance rates between detection methods

In order to evaluate the concordance between different methods, we combined both cohorts. Of the total of 539 cases, *HER2* gene amplification was significantly associated with protein overexpression (ρ, 0.601; *P*<0.001) and mRNA overtranscription (ρ, 0.626; *P*<0.001; Table 2). All the IHC 3+ cases and all the ISH score 4 cases showed *HER2* gene amplification by dual-color SISH. The concordance rate was 95.5% between dual-color SISH and IHC detection methods. When excluding equivocally immunostained cases (IHC 2+), the concordance rate was 97.7%. *HER2* mRNA overtranscription significantly correlated with protein overexpression (ρ, 0.575; *P*<0.001; Table S1).

**Table 2:** The correlation between immunohistochemistry and silver in situ hybridization for *HER2* in all CRCs of combined cohort.

*HER2*	Immunohistochemistry score					mRNA in situ hybridization score				
gene	3+	2+	1+	0	Total	4	3	2	1 & 0	Total
amplification	*N* (%)	*N* (%)	*N* (%)	*N* (%)	*N* (%)	*N* (%)	*N* (%)	*N* (%)	*N* (%)	*N* (%)
SISH +	13 (100.0)	7 (36.8)	7 (9.7)	5 (1.1)	32 (5.9)	13 (100.0)	8 (8.8)	7 (2.8)	4 (2.2)	32 (5.9)
SISH -	0 (0)	12 (63.2)	65 (90.3)	430 (98.9)	507 (94.1)	0 (0)	83 (91.2)	246 (97.2)	178 (97.8)	507 (94.1)
Total	13 (100.0)	19 (100.0)	73 (100.0)	435 (100.0)	539 (100.0)	13 (100.0)	91 (100.0)	253 (100.0)	182 (100.0)	539 (100.0)

Abbreviations: CRC, colorectal cancer; *HER2*, human epidermal growth factor receptor 2; SISH, silver in-situ hybridization; *N*, number; mRNA, messenger RNA.

All the IHC 3+ cases and all the ISH score 4 cases showed *HER2* gene amplification by dual-color SISH. The concordance rate was 95.5% between dual-color SISH and IHC detection methods.

**Figure 2:**
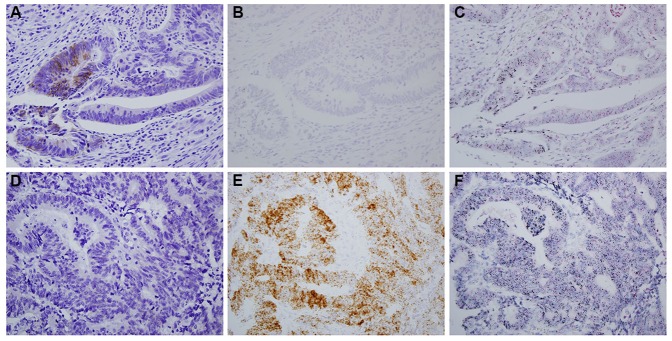
Representative figures of the cases with gene amplification and mRNA overtranscription, but without HER2 protein overexpression. (A–C) HER2 IHC 1+ in cancer focal area (A), focal mRNA overtranscription (B) and *HER2* gene amplification (C) in accordance with protein expression area. (D–F) No expression of HER2 protein (D), but diffuse mRNA overtranscription (E) and *HER2* gene amplification (F) in one case.

Of the 539 cases, 12 cases showed *HER2* gene amplification, but at IHC 0 or 1+, which is described in detail in Table S2. In one case, homogeneous *HER2* gene amplification and mRNA overtranscription were observed over the entire tumor core, but HER2 protein was not expressed in any specific area of the tumor core (Figure 2). In the other 11 cases, HER2 protein was expressed focally with an IHC 1+ score, and *HER2* gene amplification was observed localized within the protein expression area (Figure 2).

### Correlation between *HER2* status and clinicopathological features

To investigate the clinical relevance of *HER2* status, we evaluated the association between clinicopathological variables and *HER2* status. HER2 protein overexpression was not associated with MSI status or aggressive behavior including infiltrative tumour border, invasion depth, lymph node metastasis, distant metastasis, and perineural invasion (*P*>0.05; Table S3), whereas it was associated with tumor location in the rectum (*P* = 0.033 in cohort 1; Table 3). *HER2* mRNA overtranscription was not associated with any clinicopathological variables, except tumor location in the rectum (*P* = 0.001 in cohort 1, *P* = 0.026 in cohort 2; Table 3). *HER2* gene amplification was also associated with tumor location and was more frequently found in in the rectum than in the right or left colon (*P* = 0.013 in cohort 1, *P* = 0.009 in cohort 2; Table 3, Table S4).

**Table 3:** The relationship between tumor location and *HER2* status in each cohort.

*HER2* status	Primary location (cohort 1)				Primary location (cohort 2)			
	Right colon	Left colon	Rectum	*P* †	Right colon	Left colon	Rectum	*P* †
	*N* (%)	*N* (%)	*N* (%)		*N* (%)	*N* (%)	*N* (%)	
HER2 IHC				0.033				0.119
IHC 0/1+	99 (97.1)	131 (94.9)	113 (90.4)		45 (95.7)	59 (98.3)	60 (89.6)	
IHC 2+/3+	3 (2.9)	7 (5.1)	12 (9.6)		2 (4.3)	1 (1.7)	7 (10.4)	
*HER2* ISH				0.001				0.026
score 0–3	102 (100)	137 (99.3)	117 (93.6)		47 (100)	60 (100)	63 (94.0)	
score 4	0 (0)	1 (0.7)	8 (6.4)		0 (0)	0 (0)	4 (6.0)	
*HER2* SISH				0.013				0.009
not amplified	99 (97.1)	133 (96.4)	112 (89.6)		46 (97.9)	59 (98.3)	58 (86.6)	
amplified	3 (2.9)	5 (3.6)	13 (10.4)		1 (2.1)	1 (1.7)	9 (13.4)	
*HER2* positivity*				0.001				0.125
negative	102 (100)	135 (97.8)	115 (92.0)		46 (97.9)	59 (98.3)	62 (92.5)	
positive	0 (0)	3 (2.2)	10 (8.0)		1 (2.1)	1 (1.7)	5 (7.5)	
Total	102 (27.9)	138 (37.8)	125 (34.2)		47 (27.0)	60 (34.5)	67 (38.5)	

Abbreviations: *HER2*, human epidermal growth factor receptor 2; IHC, immunohistochemistry; ISH, in-situ hybridization; SISH, silver in-situ hybridization; *N*, number.

*HER2 positivity was defined as HER2 IHC 3+ and HER2 IHC 2+ with gene amplification.

† *P* values were estimated using linear-by-linear association.

To determine the prognostic significance of *HER2*-positive status, we performed survival analyses in each cohort. All the patients from cohort 1 and 2 were successfully followed up for survival analysis. The mean follow-up time was 68 months (range: 1–85 months); 98 patients (26.8%) died of cancer in cohort 1. In cohort 2, the mean follow-up time was 66 months (range: 1–105 months); 72 patients (41.4%) died of cancer. *HER2* gene amplification, mRNA overtranscription, or protein overexpression was not associated with disease-specific survival in cohort 1 and cohort 2 (*P*>0.05; Figure S1). In cohort 1, age, tumor size, tumor stage, histologic grade, lymphatic invasion, perineural invasion, venous invasion, and infiltrative tumor border were significantly associated with patients' prognosis (*P*<0.05) by univariate survival analyses. Histologic differentiation, perineural invasion, age, and tumor stage were identified as independent prognostic factors for disease-specific survival (*P*<0.05; data not shown). However, in cohort 2 cases, tumor location, tumor stage, histologic differentiation, lymphatic invasion, and perineural invasion predicted patients' prognosis by univariate survival analysis (*P*<0.05), of which tumour stage (*P* = 0.018) and lymphatic invasion (*P* = 0.024) were independent risk factors according to multivariate analysis (data not shown).

## Discussion

The purpose of this study was to identify the incidence of *HER2*-positive status in two retrospective cohorts, including consecutive CRCs and advanced CRCs with distant metastasis, and to clarify their clinical significance. Herein, we observed that *HER2* gene amplification and protein overexpression were found in about 6% of CRCs from both cohorts, CRCs with *HER2* gene amplification were located more commonly in the rectum than in the right or left colon, *HER2* status was not associated with aggressive clinicopathological features or worse prognosis, and the concordance rates between detection methods were very high. To the best of our knowledge, this is the first report to evaluate *HER2*-positive status using several techniques in a large-scale study of East Asian CRC patients.

To date, several studies have reported that the frequency of HER2 protein overexpression varies widely, from 0% to 80% in CRC [28], [29], and its prognostic significance is controversial [28], [30]–[32]. This debate might be attributed by several causes, such as primary antibody difference, a difference in scoring systems for HER2 protein expression, a difference of technical approach, sample size, racial differences, and heterogeneity of study population. When accepted staining and scoring techniques were used, the rate of membranous and cytoplasmic overexpression for HER2 were reported in approximately 5% and 30%, respectively [29]. We used accepted staining and scoring methods, and membranous overexpression of HER2 was observed in approximately 6%. These findings suggest that trastuzumab may be effective in a minority of CRC patients [33]. Alternatively, intracellular HER2-targeting compounds might be attractive treatment option in one-third of CRC patients, if cytoplasmic HER2 is really actively involved in carcinogenesis of CRC [29], [33].

It is worth noting that the concordance rate was 95.5% between dual-color SISH and IHC detection methods in the present study. When excluding equivocally immunostained cases (IHC 2+), the concordance rate was 97.7%. These concordance rates were enough to use just the IHC method for screening *HER2* gene amplification, and similar to the rates seen in gastro-esophageal cancer in the previous studies [4], [34], [35]. Among the discrepant cases, one case had *HER2* gene amplification with homogenous distribution, but HER2 protein was not expressed. This finding has been reported in gastro-esophageal cancer accounting for 2% to 33% of the cases [4], [34]–[37]. This phenomenon necessitates further studies, but the following hypothesis could explain it: erroneous post-translation processes, such as incorrect three dimensional folding, dysfunctional localization of the protein to the cell membrane, and inappropriate protein glycosylation, leading to decreased HER2 protein expression, even though the *HER2* gene is amplified [4]. In our case, *HER2* mRNA was overtranscribed with homogeneous distribution supporting the hypothesis noted above. However, despite high concordance rates between detection methods in the present study, *HER2* mRNA ISH is currently not a standard method for assessment of *HER2* status, and there has not yet been established cut-off to define mRNA overtanscription. Accordingly, further studies are necessary to validate *HER2* mRNA ISH method.

Recently, Conradi et al. [21] screened for *HER2* positivity using the same methods and criteria as ours, and reported that *HER2* positivity was observed in 12.4% of rectal biopsy samples and 26.7% of rectal resected specimen. Furthermore, *HER2* positivity in resected specimens independently correlated with prolonged cancer-specific survival in rectal cancer patients. Sclafani et al. [22] also evaluated for *HER2* positivity in high-risk, locally advanced rectal cancer patients in the EXPERT-C trial of neoadjuvant capecitabine and oxaliplatin and chemoradiotherapy (CRT) with or without cetuximab. However, in their study, *HER2* positivity was only 4.3%, and it had no association with clinicopathologic parameters and patient outcome. Our study comprised right and left colon cancers and rectal cancers; thus, we were able to analyse the frequency of *HER2* amplification and overexpression according to primary location. *HER2* amplification was most frequently found in rectal cancer than in any other primary site, and *HER2* positivity of rectal cancer was 8.0% in cohort 1 and 7.5% in cohort 2. The inconsistency for frequency of *HER2* positivity likely reflects the possible effect of several factors such as ethnicity, different study population, and tumor heterogeneity. Especially, in two previous studies, disagreement of *HER2* positivity between biopsy and resected specimen was observed [21], [22]. In light of these findings, it is suggested that intratumoral *HER2* heterogeneity may exist in CRCs, which has also been reported in studies of gastric cancer [10]. Further studies are required for clarification.

Traditionally, CEP17 alterations have affected the measurement of the *HER2*/CEP17 ratio. Consequently some cases were misclassified as non-amplified. In the present study, CEP17 polysomy was a rare event accounting for 2.6% of the cases, thus it did not induce misleading interpretation in *HER2* gene amplification. CEP17 polysomy was not clinically significant in patients with CRCs.

Although two clinical trials had tried to investigate the benefit of anti-HER2 therapy in advanced or metastatic CRC, the trials were closed prematurely due to low accrual related to the low incidence of HER2 overexpression in advanced CRC patients [38], [39]. However, one recent study showed that *HER2* served to predict resistance to anti-EGFR monoclonal antibody in patient-derived xenografts from metastatic CRC, leading the authors to suggest a possibility of combined therapies in cetuximab-resistant CRC patients [40]. Moreover, a recent HERACLES trial (HER2 Amplification for Colo-rectaL Cancer Enhanced Stratification) is currently investigating the use of trastuzumab plus lapatinib or pertuzumab in metastatic CRCs with *HER2* amplification [41]. Therefore, our findings would be helpful in developing and applying *HER2* targeted therapy in CRC patients. Because mutation results of *KRAS* and *BRAF* were not included in the present study, we were unable to elucidate relationship between *HER2* status and *KRAS* or *BRAF* in CRC. It is the concerned limitation of our study since *KRAS* and *BRAF* are very important to determine treatment approach in CRC patients. Additionally, our study has potential weaknesses, such as retrospective designed study, assessment of *HER2* using a tissue array method, heterogenous study population in a single institution, and selecting patients in cohort 2. We enrolled the CRC patients with available surgically resected cancer tissues from primary tumors in cohort 2. Not all advanced CRC patients with metastatic diseases were included and far advanced cases were not enrolled because of their inoperability. Therefore, unrecognized biases might have influenced our survival results. Furthermore, a small number of *HER2* positive tumors could preclude robust statistical analysis, thus further large-scale studies are required to validate our results.

In conclusion, *HER2* gene amplification and protein overexpression were identified in about 6% of CRC patients and has no clinical implication except in tumor location in the present study. We also demonstrated high concordance rates between IHC and dual-color SISH methods in the combined cohorts. Although a minority of CRC patients exhibited *HER2* gene amplification, these patients would be potential candidates for anti-HER2 therapy, and IHC could be primary screening test for patient selection.

## Supporting Information

Figure S1 **Kaplan-Meier survival curves according to HER2 status by each detection method.** (A–B) Survival curves according to HER2 protein expression status in cohort 1 (A) and cohort 2 (B). (C–D) Survival curves according to *HER2* mRNA expression status in cohort 1 (C) and cohort 2 (D). (E–F) Survival curves according to *HER2* gene amplification status in cohort 1 (E) and cohort 2 (F).
(JPG)

Table S1 **The relationship between immunohistochemistry and mRNA in situ hybridization for** ***HER2*** **in CRCs of combined cohort.**
(DOCX)

Table S2 **The details of the cases with** ***HER2*** **gene amplification, but IHC 0 or 1+ results.**
(DOCX)

Table S3 **The association between HER2 protein expression and clinicopathologic variables in CRCs of each cohort.**
(DOCX)

Table S4 **The association between** ***HER2*** **gene amplification and clinicopathologic factors in CRCs of each cohort.**
(DOCX)
