# Supplementary figures and images for: HER2 Status in Colorectal Cancer: Its Clinical Significance and the Relationship between HER2 Gene Amplification and Expression
Source: PLoS One. 2014 May 30;9(5):e98528. doi: 10.1371/journal.pone.0098528 (PMC4039475; doi:10.1371/journal.pone.0098528)

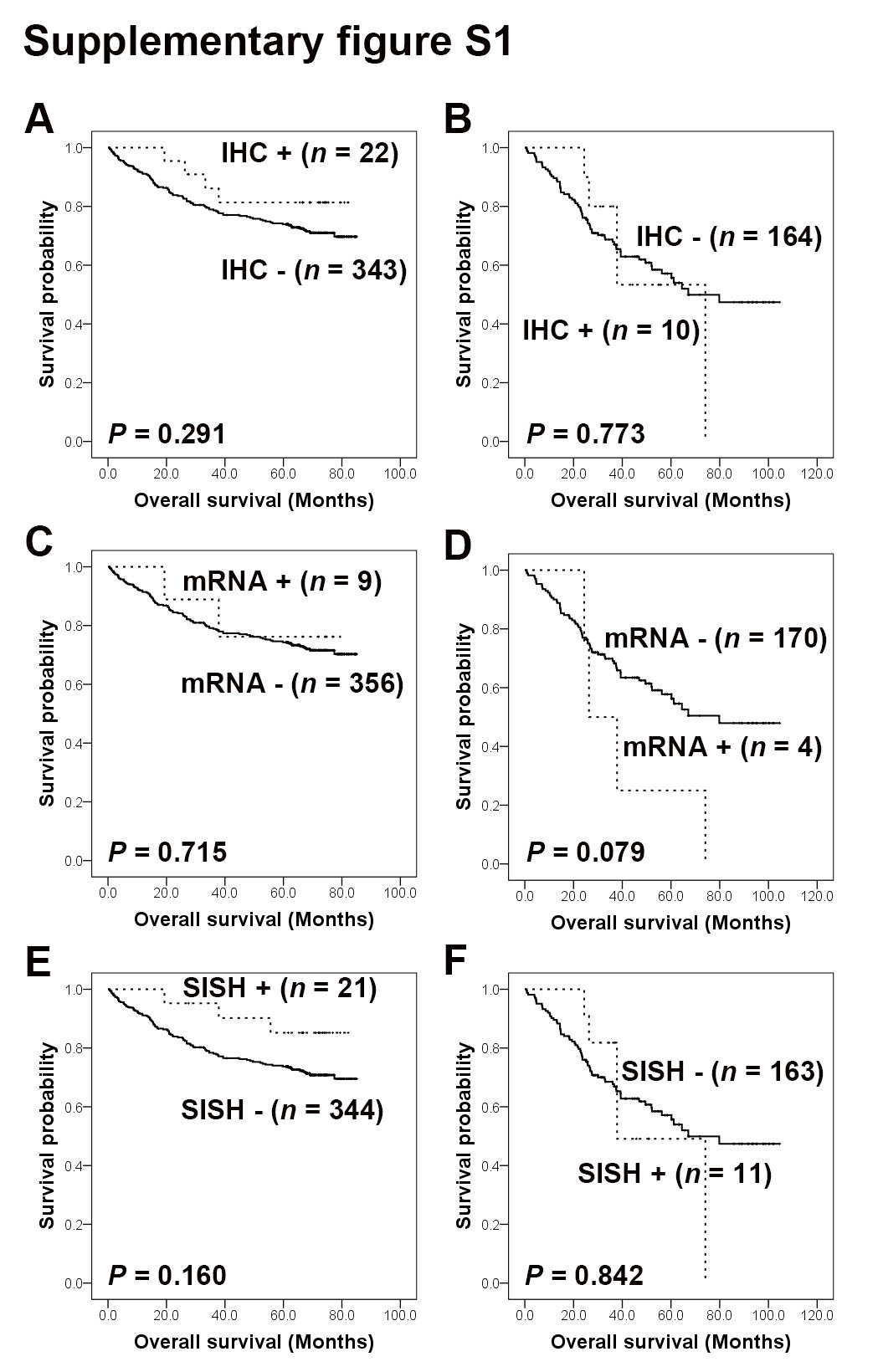

Supplement: Figure S1 — Kaplan-Meier survival curves according to HER2 status by each detection method. (A–B) Survival curves according to HER2 protein expression status in cohort 1 (A) and cohort 2 (B). (C–D) Survival curves according to HER2 mRNA expression status in cohort 1 (C) and cohort 2 (D). (E–F) Survival curves according to HER2 gene amplification status in cohort 1 (E) and cohort 2 (F). (JPG) [file pone.0098528.s001.jpg]
