# Supplementary material for: HER2 Status in Colorectal Cancer: Its Clinical Significance and the Relationship between HER2 Gene Amplification and Expression
Source: PLoS One. 2014 May 30;9(5):e98528. doi: 10.1371/journal.pone.0098528 (PMC4039475; doi:10.1371/journal.pone.0098528)
Supplement: Table S1 — The relationship between immunohistochemistry and mRNA in situ hybridization for HER2 in CRCs of combined cohort. (DOCX) [file pone.0098528.s002.docx]

**Table S1.** The relationship between immunohistochemistry and mRNA in situ hybridization for *HER2* in CRCs of combined cohort

| HER2 | Immunohistochemistry score | | | | |
| --- | --- | --- | --- | --- | --- |
| mRNA | 0 | 1+ | 2+ | 3+ | Total |
| ISH score | *N* (%) | *N* (%) | *N* (%) | *N* (%) | *N* (%) |
| 0 | 22 (5.1) | 1 (1.4) | 0 (0) | 0 (0) | 23 (4.3) |
| 1 | 137 (31.5) | 18 (25.0) | 4 (21.1) | 0 (0) | 159 (29.5) |
| 2 | 217 (49.9) | 32 (44.4) | 4 (21.1) | 0 (0) | 253 (46.9) |
| 3 | 58 (13.3) | 21 (29.2) | 11 (57.9) | 1 (7.7) | 91 (16.9) |
| 4 | 1 (0.2) | 0 (0) | 0 (0) | 12 (92.3) | 13 (2.4) |
| Total | 435 (100) | 72 (100) | 19 (100) | 13 (100) | 539 (100) |

Abbreviations: *HER2*, human epidermal growth factor receptor 2; CRC, colorectal cancer; ISH, in-situ hybridization; N, number.
