# Supplementary material for: HER2 Status in Colorectal Cancer: Its Clinical Significance and the Relationship between HER2 Gene Amplification and Expression
Source: PLoS One. 2014 May 30;9(5):e98528. doi: 10.1371/journal.pone.0098528 (PMC4039475; doi:10.1371/journal.pone.0098528)
Supplement: Table S2 — The details of the cases with HER2 gene amplification, but IHC 0 or 1+ results. (DOCX) [file pone.0098528.s003.docx]

**Table S2.** The details of the cases with *HER2* gene amplification, but IHC 0 or 1+ results

| Case No. | SISH | | |  |  | mRNA ISH |  | IHC | | |
| --- | --- | --- | --- | --- | --- | --- | --- | --- | --- | --- |
|  | Ratio | *HER2* GCN | CEP17 CN | distribution |  | Score |  | Intensity | Proportion | staining pattern |
| 151245 | 10.45 | 24.55 | 2.35 | Diffuse |  | 4 |  | 0 | 0 | No reactivity |
| 151337 | 2.18 | 5.45 | 2.50 | Focal |  | 1 |  | 1 | 1% | Base |
| 190612 | 2.27 | 4.70 | 2.08 | Focal |  | 2 |  | 1 | 5% | Base |
| 190624 | 3.19 | 6.78 | 2.13 | Focal |  | 2 |  | 1 | 1% | Base |
| 190634 | 2.12 | 4.87 | 2.30 | Focal |  | 2 |  | 1 | 6% | Incomplete lateral |
| 190808 | 2.29 | 4.80 | 2.10 | Focal |  | 2 |  | 1 | 5% | Incomplete lateral |
| 190816 | 2.18 | 4.87 | 2.23 | Focal |  | 3 |  | 1 | 1% | Base |
| 190934 | 2.39 | 5.03 | 2.10 | Focal |  | 1 |  | 1 | 1% | Incomplete lateral |
| 157943 | 2.64 | 5.15 | 1.95 | Focal |  | 2 |  | 1 | 5% | Incomplete lateral |
| 157960 | 3.63 | 5.80 | 1.60 | Focal |  | 2 |  | 1 | 1% | Incomplete lateral |
| 158020 | 2.62 | 5.10 | 1.95 | Focal |  | 0 |  | 1 | 5% | Incomplete lateral |
| 158028 | 2.50 | 5.75 | 2.30 | Focal |  | 2 |  | 1 | 5% | Incomplete lateral |

Abbreviations: *HER2*, human epidermal growth factor receptor 2; SISH, silver in-situ hybridization; ISH, in situ hybridization; IHC, immunohistochemistry; GCN, gene copy number.
