# Supplementary material for: HER2 Status in Colorectal Cancer: Its Clinical Significance and the Relationship between HER2 Gene Amplification and Expression
Source: PLoS One. 2014 May 30;9(5):e98528. doi: 10.1371/journal.pone.0098528 (PMC4039475; doi:10.1371/journal.pone.0098528)
Supplement: Table S3 — The association between HER2 protein expression and clinicopathologic variables in CRCs of each cohort. (DOCX) [file pone.0098528.s004.docx]

**Table S3.** The association between HER2 protein expression and clinicopathologic variables in CRCs of each cohort

| Characteristic | Cohort 1 | | | |  | Cohort 2 | | | |
| --- | --- | --- | --- | --- | --- | --- | --- | --- | --- |
|  | Total | *HER2* protein overexpression | |  |  | Total | *HER2* protein overexpression | |  |
|  |  | Negative  (0 & 1+) | Positive  (2+ & 3+) | *P*† |  |  | Negative  (0 & 1+) | Positive  (2+ & 3+) | *P*† |
|  | *N* (%) | *N* (%) | *N* (%) |  |  | *N* (%) | *N* (%) | *N* (%) |  |
| Age (years) |  |  |  | 0.783 |  |  |  |  | 0.133 |
| Median | 65.0 | 65.0 | 66.0 |  |  | 60.0 | 60.0 | 53.5 |  |
| Range | 20 to 95 | 20 to 95 | 37 to 74 |  |  | 28 to 93 | 28 to 93 | 30 to 71 |  |
| Gender |  |  |  | 0.715 |  |  |  |  | 0.346 |
| Male | 202 (55.3) | 189 (93.6) | 13 (6.4) |  |  | 94 (54.0) | 87 (92.6) | 7 (7.4) |  |
| Female | 163 (44.7) | 154 (94.5) | 9 (5.5) |  |  | 80 (46.0) | 77 (96.3) | 3 (3.8) |  |
| Histologic differentiation |  |  |  | 1.000 |  |  |  |  | 0.638 |
| Low grade | 331 (90.7) | 311 (94.0) | 20 (6.0) |  |  | 149 (85.6) | 141 (94.6) | 8 (5.4) |  |
| High grade | 34 (9.3) | 32 (94.1) | 2 (5.9) |  |  | 25 (14.4) | 23 (92.0) | 2 (8.0) |  |
| Tumor border |  |  |  | 0.374 |  |  |  |  | 1.000 |
| Expanding | 59 (16.2) | 54 (91.5) | 5 (8.5) |  |  | 15 (8.6) | 14 (93.3) | 1 (6.7) |  |
| Infiltrative | 306 (83.8) | 289 (94.4) | 17 (5.6) |  |  | 159 (91.4) | 150 (94.3) | 9 (5.7) |  |
| Tumor size (cm) |  |  |  | 0.343 |  |  |  |  | 0.861 |
| Median | 5.0 | 5.0 | 4.4 |  |  | 5.4 | 5.3 | 5.5 |  |
| Range | 0 to 13.0 | 1.0 to 13.0 | 1.7 to 10.8 |  |  | 2.0 to 27.0 | 2.0 to 27.0 | 3.0 to 7.0 |  |
| Tumor depth (pT) |  |  |  | 0.173 |  |  |  |  | 0.382 |
| 1 | 14 (3.8) | 14 (100) | 0 (0) |  |  | 1 (0.6) | 1 (100) | 0 (0) |  |
| 2 | 46 (12.6) | 39 (84.8) | 7 (15.2) |  |  | 4 (2.3) | 4 (100) | 0 (0) |  |
| 3 | 238 (65.2) | 225 (94.5) | 13 (5.5) |  |  | 102 (58.6) | 97 (95.1) | 5 (4.9) |  |
| 4 | 67 (18.4) | 65 (97.0) | 2 (3.0) |  |  | 67 (38.5) | 62 (92.5) | 5 (7.5) |  |
| LN metastasis |  |  |  | 0.913 |  |  |  |  | 0.691 |
| Absent | 170 (46.6) | 160 (94.1) | 10 (5.9) |  |  | 32 (18.4) | 31 (96.9) | 1 (3.1) |  |
| Present | 195 (53.4) | 183 (93.8) | 12 (6.2) |  |  | 142 (81.6) | 133 (93.7) | 9 (6.3) |  |
| Lymphatic invasion |  |  |  | 0.811 |  |  |  |  | 0.739 |
| Absent | 157 (43.0) | 147 (93.6) | 10 (6.4) |  |  | 60 (34.5) | 56 (93.3) | 4 (6.7) |  |
| Present | 208 (57.0) | 196 (94.2) | 12 (5.8) |  |  | 114 (65.5) | 108 (94.7) | 6 (5.3) |  |
| Perineural invasion |  |  |  | 0.905 |  |  |  |  | 1.000 |
| Absent | 253 (69.3) | 238 (94.1) | 15 (5.9) |  |  | 84 (48.3) | 79 (94.0) | 5 (6.0) |  |
| Present | 112 (30.7) | 105 (93.8) | 7 (6.3) |  |  | 90 (51.7) | 85 (94.4) | 5 (5.6) |  |
| Venous invasion |  |  |  | 0.778 |  |  |  |  | 0.503 |
| Absent | 297 (81.4) | 278 (93.6) | 19 (6.4) |  |  | 120 (69.0) | 114 (95.0) | 6 (5.0) |  |
| Present | 68 (18.6) | 65 (95.6) | 3 (4.4) |  |  | 54 (31.0) | 50 (92.6) | 4 (7.4) |  |
| Distant metastasis at diagnosis |  |  |  | 0.777 |  |  |  |  | 1.000 |
| Absent | 299 (81.9) | 280 (93.6) | 19 (6.4) |  |  | 61 (35.1) | 58 (95.1) | 3 (4.9) |  |
| Present | 66 (18.1) | 63 (95.5) | 3 (4.5) |  |  | 113 (64.9) | 106 (93.8) | 7 (6.2) |  |
| TNM stage |  |  |  | 0.206 |  |  |  |  | 0.409 |
| I | 46 (12.6) | 40 (87.0) | 6 (13.0) |  |  | 4 (2.3) | 4 (100) | 0 (0) |  |
| II | 118 (32.3) | 113 (95.8) | 5 (4.2) |  |  | 17 (9.8) | 17 (100) | 0 (0) |  |
| III | 135 (37.0) | 127 (94.1) | 8 (5.9) |  |  | 40 (23.0) | 37 (92.5) | 3 (7.5) |  |
| IV | 66 (18.1) | 63 (95.5) | 3 (4.5) |  |  | 113 (64.9) | 106 (93.8) | 7 (6.2) |  |
| MSI analysis |  |  |  |  |  |  |  |  |  |
| MSS/MSI-L | 321 (90.9) | 301 (90.9) | 20 (90.9) | 1.000 |  | 159 (98.1) | 149 (98.0) | 10 (100.0) | 1.000 |
| MSI-H | 32 (9.1) | 30 (9.1) | 2 (9.1) |  |  | 3 (1.9) | 3 (2.0) | 0 (0) |  |

Abbreviations: CRC, colorectal cancer; N, number; LN, lymph node; TNM, tumor-node- metastasis; MSI, microsatellite instability; MSS, microsatellite stability; MSI-L, microsatellite instability-low; MSI-H, microsatellite instability-high.

† *P* values were estimated using χ^2^ test, Fisher’s exact test, linear-by-linear association, or Mann-Whitney U test.

‡ For statistical convenience, T stage and TNM stage were analyzed as dichotomous covariate (low versus high).
