# Supplementary material for: HER2 Status in Colorectal Cancer: Its Clinical Significance and the Relationship between HER2 Gene Amplification and Expression
Source: PLoS One. 2014 May 30;9(5):e98528. doi: 10.1371/journal.pone.0098528 (PMC4039475; doi:10.1371/journal.pone.0098528)
Supplement: Table S4 — The association between HER2 gene amplification and clinicopathologic factors in CRCs of each cohort. (DOCX) [file pone.0098528.s005.docx]

**Table S4.** The association between *HER2* gene amplification and clinicopathologic factors in CRCs of each cohort

| Characteristic | Cohort 1 | | | |  | Cohort 2 | | | |
| --- | --- | --- | --- | --- | --- | --- | --- | --- | --- |
|  | Total | *HER2* amplification* | |  |  | Total | *HER2* amplification* | |  |
|  |  | Negative | Positive | *P*† |  |  | Negative | Positive | *P*† |
|  | *N* (%) | *N* (%) | *N* (%) |  |  | *N* (%) | *N* (%) | *N* (%) |  |
| Age (years) |  |  |  | 0.886 |  |  |  |  | 0.078 |
| Median | 65.0 | 65.0 | 65.5 |  |  | 60.0 | 60.0 | 55.0 |  |
| Range | 20.0 to 95.0 | 20.0 to 95.0 | 47.0 to 74.0 |  |  | 28 to 93 | 28.0 to 93.0 | 30.0 to 71.0 |  |
| Gender |  |  |  | 0.779 |  |  |  |  | 0.971 |
| Male | 202 (55.3) | 191 (94.6) | 11 (5.4) |  |  | 94 (54.0) | 88 (93.6) | 6 (6.4) |  |
| Female | 163 (44.7) | 153 (93.9) | 10 (6.1) |  |  | 80 (46.0) | 75 (93.8) | 5 (6.3) |  |
| Histologic differentiation |  |  |  | 1.000 |  |  |  |  | 0.055 |
| Low grade | 331 (90.7) | 312 (94.3) | 19 (5.7) |  |  | 149 (85.6) | 142 (95.3) | 7 (4.7) |  |
| High grade | 34 (9.3) | 32 (94.1) | 2 (5.9) |  |  | 25 (14.4) | 21 (84.0) | 4 (16.0) |  |
| Tumor border |  |  |  | 1.000 |  |  |  |  | 1.000 |
| Expanding | 59 (16.2) | 56 (94.9) | 3 (5.1) |  |  | 15 (8.6) | 14 (93.3) | 1 (6.7) |  |
| Infiltrative | 306 (83.8) | 288 (94.1) | 18 (5.9) |  |  | 159 (91.4) | 149 (93.7) | 10 (6.3) |  |
| Tumor size |  |  |  | 0.872 |  |  |  |  | 0.740 |
| Median | 5.0 | 5.0 | 5.2 |  |  | 5.4 | 5.3 | 5.8 |  |
| Range | 0 to 13.0 | 0 to 13.0 | 2.1 to 9.5 |  |  | 2.0 to 27.0 | 2.0 to 27.0 | 3.0 to 10.0 |  |
| Tumor depth (pT) |  |  |  | 0.893 |  |  |  |  | 0.936 |
| 1 | 14 (3.8) | 14 (100.0) | 0 (0) |  |  | 1 (0.6) | 1 (100.0) | 0 (0) |  |
| 2 | 46 (12.6) | 41 (89.1) | 5 (10.9) |  |  | 4 (2.3) | 4 (100.0) | 0 (0) |  |
| 3 | 238 (65.2) | 226 (95.0) | 12 (5.0) |  |  | 102 (58.6) | 95 (93.1) | 7 (6.9) |  |
| 4 | 67 (18.4) | 63 (94.0) | 4 (6.0) |  |  | 67 (38.5) | 63 (94.0) | 4 (6.0) |  |
| LN metastasis |  |  |  | 0.725 |  |  |  |  | 0.099 |
| Absent | 170 (46.6) | 161 (94.7) | 9 (5.3) |  |  | 32 (18.4) | 32 (100.0) | 0 (0) |  |
| Present | 195 (53.4) | 183 (93.8) | 12 (6.2) |  |  | 142 (81.6) | 131 (92.3) | 11 (7.7) |  |
| Lymphatic invasion |  |  |  | 0.661 |  |  |  |  | 0.750 |
| Absent | 157 (43.0) | 147 (93.6) | 10 (6.4) |  |  | 60 (34.5) | 57 (95.0) | 3 (5.0) |  |
| Present | 208 (57.0) | 197 (94.7) | 11 (5.3) |  |  | 114 (65.5) | 106 (93.0) | 8 (7.0) |  |
| Perineural invasion |  |  |  | 0.786 |  |  |  |  | 0.847 |
| Absent | 253 (69.3) | 239 (94.5) | 14 (5.5) |  |  | 84 (48.3) | 79 (94.0) | 5 (6.0) |  |
| Present | 112 (30.7) | 105 (93.8) | 7 (6.3) |  |  | 90 (51.7) | 84 (93.3) | 6 (6.7) |  |
| Venous invasion |  |  |  | 0.777 |  |  |  |  | 0.740 |
| Absent | 297 (81.4) | 279 (93.9) | 18 (6.1) |  |  | 120 (69.0) | 113 (94.2) | 7 (5.8) |  |
| Present | 68 (18.6) | 65 (95.6) | 3 (4.4) |  |  | 54 (31.0) | 50 (92.6) | 4 (7.4) |  |
| Distant metastasis at diagnosis |  |  |  | 0.778 |  |  |  |  | 0.333 |
| Absent | 299 (81.9) | 281 (94.0) | 18 (6.0) |  |  | 61 (35.1) | 59 (96.7) | 2 (3.3) |  |
| Present | 66 (18.1) | 63 (95.5) | 3 (4.5) |  |  | 113 (64.9) | 104 (92.0) | 9 (8.0) |  |
| TNM stage |  |  |  | 0.677 |  |  |  |  | 0.162 |
| I | 46 (12.6) | 42 (91.3) | 4 (8.7) |  |  | 4 (2.3) | 4 (100.0) | 0 (0) |  |
| II | 118 (32.3) | 113 (95.8) | 5 (4.2) |  |  | 17 (9.8) | 17 (100.0) | 0 (0) |  |
| III | 135 (37.0) | 126 (93.3) | 9 (6.7) |  |  | 40 (23.0) | 38 (95.0) | 2 (5.0) |  |
| IV | 66 (18.1) | 63 (95.5) | 3 (4.5) |  |  | 113 (64.9) | 104 (92.0) | 9 (8.0) |  |
| MSI analysis |  |  |  | 1.000 |  |  |  |  | 1.000 |
| MSS/MSI-L | 321 (90.9) | 302 (90.7) | 19 (95.0) |  |  | 157 (98.1) | 146 (98.0) | 11 (100.0) |  |
| MSI-H | 32 (9.1) | 31 (9.3) | 1 (5.0) |  |  | 3 (1.9) | 3 (2.0) | 0 (0) |  |

Abbreviations: CRC, colorectal cancer; N, number; LN, lymph node; TNM, tumor-node- metastasis; IHC, immunohistochemistry; MSI, microsatellite instability; MSS, microsatellite stability; MSI-L, microsatellite instability-low; MSI-H, microsatellite instability-high.

* *HER2*:CEP17 ratio ≥2.0 regarded as *HER2* gene amplification.

† *P* values were estimated using χ^2^ test, Fisher’s exact test, linear-by-linear association or Wilcoxon/Mann-Whitney U test

‡ For statistical convenience, T stage and TNM stage were analyzed as dichotomous covariate (low versus high).
